# Supplementary material for: Role of Probiotics in Preventing Carbapenem-Resistant Enterobacteriaceae Colonization in the Intensive Care Unit: Risk Factors and Microbiome Analysis Study
Source: Microorganisms. 2023 Dec 12;11(12):2970. doi: 10.3390/microorganisms11122970 (PMC10745884; doi:10.3390/microorganisms11122970)
Supplement: Supplementary file 1 [file microorganisms-11-02970-s001.zip › Supplementary_Figure S2.pdf]

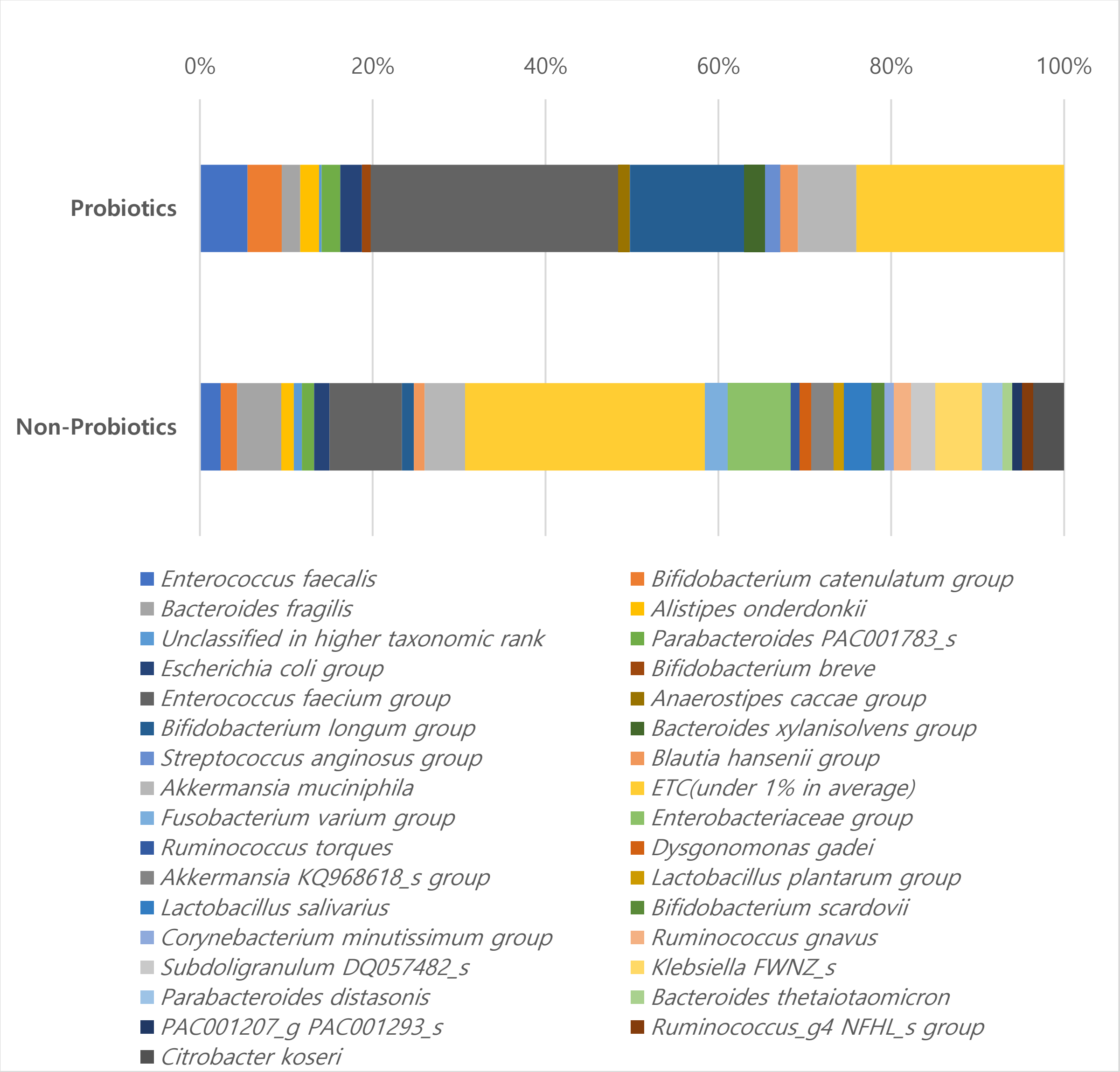

Supplementary Figure S2. Comparison of species abundance in patients colonized with carbapenem-resistant *Enterobacteriaceae* according to probiotics administration.
