# Supplementary material for: Role of Probiotics in Preventing Carbapenem-Resistant Enterobacteriaceae Colonization in the Intensive Care Unit: Risk Factors and Microbiome Analysis Study
Source: Microorganisms. 2023 Dec 12;11(12):2970. doi: 10.3390/microorganisms11122970 (PMC10745884; doi:10.3390/microorganisms11122970)
Supplement: Supplementary file 1 [file microorganisms-11-02970-s001.zip › Supplementary_Figure S3.pdf]

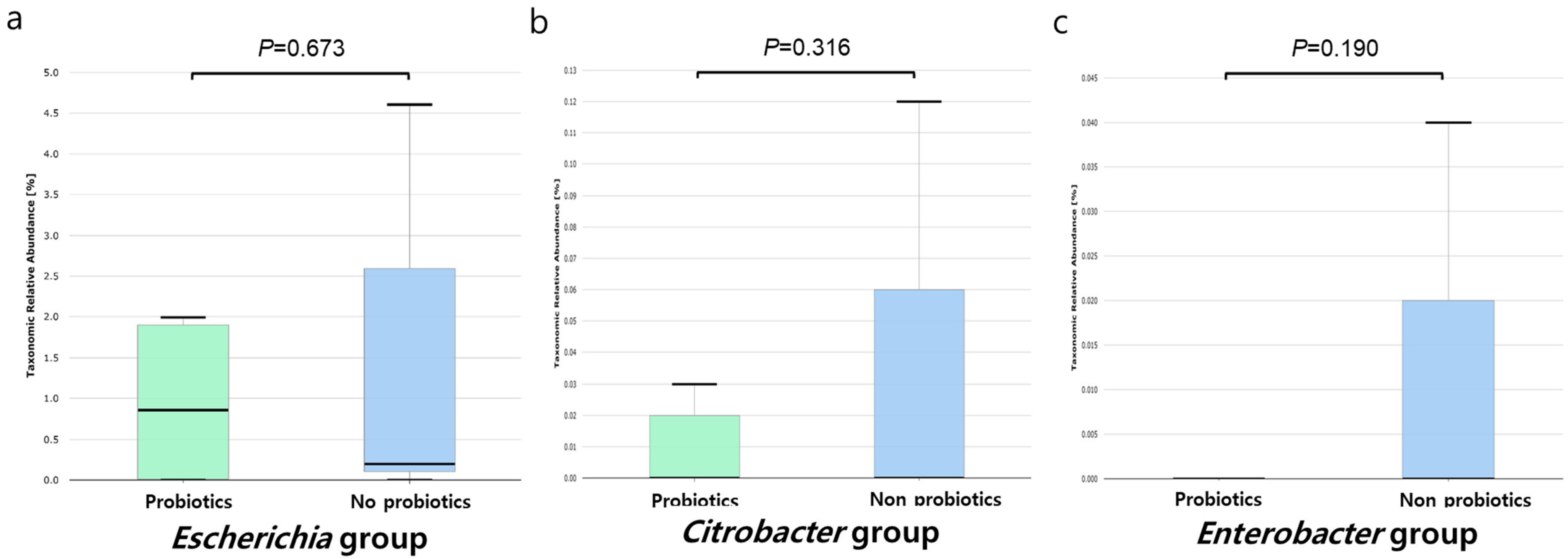

Supplementary Figure S3 Microbiome analysis for patients with carbapenem-resistant *Enterobacteriaceae* colonization according to probiotics administration. a. relative abundance of *Escherichia* family between the probiotics and non-probiotics groups. b. relative abundance of *Citrobacter* genus between the probiotics and non-probiotics groups. c. relative abundance of *Enterobacter* genus between the probiotics and non-probiotics groups

Abbreviation: CRE, carbapenem-resistant *Enterobacteriaceae.*
